# Regulatory Mechanisms of Total Soluble Solids in Tomato: From QTL Mapping to Gene Editing

**DOI:** 10.3390/foods14213692

**Published:** 2025-10-29

**Authors:** Minghua Xu, Shujing Ji, Shengqun Pang, Yongen Lu, Shouming Li, Wei Xu

**Affiliations:** 1 Key Laboratory of Special Fruits and Vegetables Cultivation Physiology and Germplasm Resources Utilization (Xinjiang Production and Construction Crops), College of Agriculture, Shihezi University, Shihezi 832003, China; xuminghua@xjshzu.com (M.X.); jishujing129@163.com (S.J.); pangshqok@shzu.edu.cn (S.P.); 2 National Key Laboratory for Germplasm Innovation and Utilization of Horticultural Crops, Huazhong Agricultural University, Wuhan 430070, China; luyongen@mail.hzau.edu.cn; 3 Facility Horticulture Research Institute, Shihezi Academy of Agriculture Science, Shihezi 832003, China

**Keywords:** total soluble solids, QTL mapping, sugar and acid metabolism, environmental factors, genome editing

## Abstract

Total Soluble Solids (TSS) in tomatoes is a core indicator for evaluating fruit quality and processing characteristics. Its composition mainly consists of soluble sugars (such as fructose and glucose) and organic acids (such as citric acid and malic acid). The contents of sugars and acids and their ratio directly affect the flavor and nutritional value. Cultivated tomatoes have a TSS of 4–6%, compared with 10–15% in wild varieties. In recent years, with the advancement of molecular biology and genomics technologies, significant progress has been made in the research on the regulatory mechanisms of tomato fruit TSS and major sugars and acids, including the identification of major quantitative trait locus (QTLs) (*Lin5*, *SlALMT9*), functional characterization via CRISPR/Cas9 and elucidation of the transporter network. Breaking the negative correlation between TSS and yield remains a major bottleneck in breeding. Analyzing the mechanism by which environmental factors regulate the TSS and optimizing cultivation measures are crucial for increasing the TSS content in tomatoes. The deep integration of cutting-edge technologies (such as Genome-wide association studies (GWAS), metabolome-wide association studies (mGWAS), Genomic selection (GS), genome editing, and crop modeling) with design breeding is expected to accelerate the development of high-TSS tomato varieties. This paper reviews the current research status from the following four aspects: QTL mapping related to tomato TSS and mining of major genes, metabolic and transport mechanisms of major sugars and acids and key genes, the influence of environmental factors on TSS, and application of genetic improvement strategies and technologies.

## 1. Introduction

Tomato (*Solanum lycopersicum*) occupies a central role in the horticultural industry. From wild species to cherry tomatoes and subsequently to large-fruited cultivars, the processes of domestication and varietal improvement have been continuous [1]. For decades, breeding programs have mainly emphasized high yield and stress resistance, while fruit quality has been consistently neglected [2]. In the past decade, people’s consumption has been accelerating the shift from “eating enough” to “eating well” and a “nutritious and healthy diet”. Consumers’ demand for high—quality fruits has been increasing day by day, and fruit quality has become the focus of breeding [3]. In tomato, TSS, consisting mainly of soluble sugars and organic acids, are one of the most important indicators of fruit quality. The distinctive flavor of tomatoes is derived from the combined action of soluble sugars, organic acids, and volatile organic compounds, with the contents and ratios of sugars and acids exerting direct control over fruit taste [4,5]. In tomato raw material processing, TSS is a key indicator determining quality and competitiveness [6]. Based on a 5% TSS threshold in tomato, for every 1% increase, it is equivalent to a 25% reduction in raw material consumption [7]. Consequently, the processing tomato market has clear quantitative minimum requirements for TSS, aiming to pursue extremely high TSS to optimize production costs. In contrast, the fresh—market tomato market focuses more on the coordination between TSS and acidity, striving to provide consumers with the best flavor experience.

Tomato TSS represents a complex quantitative trait controlled by both genetic and environmental factors, and its genetic basis has been extensively investigated. QTL mapping widely employed in tomato breeding has provided support for dissecting the genetic architecture of complex traits, facilitating marker-assisted selection and revealing molecular mechanisms. GWAS have been recognized as an approach based on linkage disequilibrium and have recently provided effective strategies for rapidly identifying QTLs that regulate TSS [8]. Wild tomato species generally exhibit TSS values of 10–15%, approximately two to three times higher than those of cultivated varieties, rendering them valuable germplasms for breeding high-TSS cultivars and mapping TSS-related QTLs [9].

TSS is primarily composed of soluble sugars and organic acids, which constitute approximately 60% of tomato dry weight. The soluble sugars mainly include glucose, fructose, and sucrose, whereas the predominant organic acids are citric acid (CA) and malic acid (MA) [10]. Sugar accumulation and acid balance are regulated by complex interactions between genetic and environmental factors. Sucrose metabolism plays a central role in sugar accumulation, with sucrose phosphate synthase (SPS), sucrose synthase (SuSy), and invertase (INV) acting as key enzymes [11,12,13]. MA and CA participate in multiple metabolic pathways, including respiration, the tricarboxylic acid (TCA) cycle, and the glyoxylate cycle, and can also undergo transformation in mitochondria through the TCA cycle [14,15]. Owing to the involvement of multiple enzymes and transcription factors, sugar-acid metabolism remains difficult to predict and regulate. With the detailed functional analysis of key genes, elucidation of molecular regulatory networks, and construction of regulatory models, it becomes possible not only to enhance sugar and acid accumulation but also to adjust their ratios. Such advances have created the potential for breeding high-quality tomato varieties with elevated TSS and improved fruit flavors. Beyond genetic factors, environmental conditions, including temperature, light, water, fertilizer application, and air composition, exert major effects on TSS. Hence, the optimization of cultivation practices is critical for enhancing tomato TSS.

Cross-breeding in tomato improvement is significantly hindered by the polygenic nature of TSS, which leads to long breeding cycles, low efficiency, and the persistent challenge of linkage drag [16]. Decades of breeding have only achieved marginal TSS benefits while sacrificing the yield. In addition, the loci controlling TSS are located in close proximity to those regulating fruit size, and a negative correlation exists between these two traits [17]. Therefore, strategies for increasing tomato TSS without substantially reducing fruit size remain a critical focus in high-quality breeding programs. Modern biotechnological approaches have provided new opportunities. Based on the identification of TSS-associated markers such as simple sequence repeats (SSR) and single nucleotide polymorphisms (SNP), molecular marker-assisted selection (MAS) has accelerated breeding efficiency [18]. GS further expands the potential of molecular markers by employing genome-wide marker data to construct predictive models, thereby enabling more accurate evaluation of breeding values, enhancing selection efficiency, and accelerating genetic gains [19]. The development of genome editing technologies, including CRISPR/Cas9, has enabled precise gene knockout, knock-in, and base editing in the tomato genome. The first commercialized genome-edited crop, the “Sicilian Rouge High GABA” tomato, has significantly increased the γ-aminobutyric acid (GABA) content in its fruits by editing the *SlGAD2/3* genes [20]. In addition, editing the promoter allows for the precise regulation of gene expression levels, providing a more powerful tool for crop breeding. These tools facilitate the creation of novel germplasms, shorten breeding cycles, and overcome interspecific hybridization barriers. On this basis, design-based breeding systematically integrates genomics, bioinformatics, and genetic design principles to achieve the comprehensive analysis and precise assembly of breeding objectives, promoting the shift from “empirical screening” to “directed design” and improving both the predictability and efficiency in tomato breeding.

Meanwhile, the application of high-throughput phenomics and artificial intelligence technologies has provided strong support for the optimization of cultivation management. By integrating multidimensional environmental data with plant phenotypic information, association models linking growth, environment, and traits can be established, offering a precise decision-making basis for water and fertilizer regulation as well as light and temperature management. These approaches maximize the TSS potential of tomato varieties and enable full-process optimization from genetic regulation to field management.

In recent years, the significance of tomato fruit quality has gained widespread recognition. However, a comprehensive review of TSS, a crucial determinant of tomato fruit quality, remains lacking. Therefore, this review article provides a comprehensive overview of research on tomato TSS. We first explore QTL mapping and gene identification, and subsequently summarize the metabolic and transport mechanisms of major sugars and acids along with their key genes. In addition, the impact of environmental factors on TSS is introduced. The article concludes by projecting breeding strategies enhanced by advanced technologies, thus offering a theoretical framework for targeted breeding of high-quality tomatoes.

## 2. Mapping and Identification of QTLs Associated with Tomato TSS

### 2.1. QTL Mapping and Identification Based on Hybrid Population

Over the past three decades, researchers have mapped more than 50 TSS QTLs on over 12 tomato chromosomes using different parental populations. The major loci are located on chromosomes 2, 3, 5, and 9 [10,21,22,23,24]. Paterson et al. mapped seven QTLs associated with fruit TSS in progeny derived from a cross between cultivated tomato (*Lycopersicon esculentum* Mill.) and its wild relative *Lycopersicon cheesmanii* [25]. Tanksley et al. identified three TSS-related QTLs, ssc5.1, ssc5.2, and ssc5.3, on chromosome 5 in approximately 170 backcross second-generation (BC2) plants derived from a cross between an elite processing inbred line and the wild species *Lycopersicon pimpinellifolium* (LA1589) [26]. Fulton et al. reported the detection of two TSS-related QTLs in a backcross population generated from cultivated tomato (*Lycopersicon esculentum* cv. ‘E6203’) and its wild relative *Lycopersicon peruvianum* [27].

Goldman et al. identified 12 TSS-related QTLs in a recombinant inbred line (RIL) population developed from a cross between cultivated tomato *Lycopersicon esculentum* and related wild tomato *Lycopersicon cheesmanii* [28]. Bernacchi et al. mapped seven QTLs associated with TSS in a near-isogenic line (NIL) population constructed using *Lycopersicon hirsutum* (LA1777) and *Lycopersicon pimpinellifolium* (LA1589) [29]. Furthermore, Bernacchi et al. identified five TSS-related QTLs located on chromosomes 3, 5, 6, and 9 in a *Lycopersicon hirsutum* introgression line population [30]. Causse et al. [23] employed a population of 75 introgression lines (ILs) with the genetic background of cultivated tomato (*Lycopersicon esculentum*, M82), each line containing a chromosomal segment introgressed from the wild species *Lycopersicon pennellii* (LA0716). They mapped multiple QTLs associated with sugar and organic acid content [23].

Among the identified QTLs, several key genes have been cloned and functionally characterized. In 1988, a comparison of carbohydrate changes and related enzyme activities during fruit development between *Lycopersicon chmielewskii* (LA1028) and cultivated tomato UC-82B revealed that LA1028 fruit accumulated sucrose [31]. Sucrose accumulation is primarily associated with reduced acid invertase (AI) activity [32]. Through gene mapping with restriction fragment length polymorphism (RFLP) and isozyme markers, a major QTL, sucrose accumulator (Sucr), was identified on chromosome 3, co-segregating with the TG102 marker and encoding vacuolar invertase [33]. Levin et al. discovered another QTL, Fgr on chromosome 4 of *Lycopersicon hirsutum*, which increased fructose content and reduced the glucose content in mature fruits without affecting the total hexose concentration [34]. To further explore the trait differences between cultivated and wild tomatoes, Eshed et al. developed an introgression line IL9-2-5 with high TSS by crossing *Lycopersicon pennellii* (LA0716) with cultivated tomato M82 [35]. Subsequently, the major QTL for TSS, Brix9-2-5 was identified, encoding the extracellular invertase *Lin5* [36]. Further research indicates that the polymorphism near the catalytic site of *Lin5* enhances invertase activity, resulting in an 11–25% increase in sugar content without reducing the total yield [37]. In the red fruits of the Lin5-silenced lines, the glucose content decreased by approximately 30–33% and the fructose content decreased by about 17–26% compared to the wild type [38], whereas Knocking out its invertase inhibitor gene can enhance invertase activity. The glucose content in CRISPR-invinh1 mutants increased by 36.39–40.82%, the fructose content increased by 35.69–42.76%, and TSS increased by 30.17–32.76% compared with the wild type [39].

In recent years, QTLs related to MA and CA contents in tomato fruits have been successively identified. Researchers have evaluated the main sugars and organic acids influencing tomato flavor in *Solanum pimpinellifolium* and its backcross inbred lines. The study revealed that *Solanum pimpinellifolium* harbored alleles that increased glucose and fructose contents and regulated acidity through changes in MA and CA levels. Compared with RFLPs/isoenzymes, SNPs have become the cornerstone of modern genetic research by virtue of their absolute advantages of high density, high throughput, and digitization. Using SNP markers, 71 QTLs related to organic acids have been identified [40]. For MA, loci ma1.2, ma1.3, ma2.1, and ma7.1 were consistently detected across populations, indicating the presence of major genetic variations related to MA content. QTLs cca2.1, ca-5E, cca6.3, cca7.1, cca8.1, ca-9F, and ca-10B may represent the major determinants of CA accumulation [41]. Tian et al. [42] used a recombinant inbred line population constructed from the cultivated tomato (*Solanum lycopersicum* ‘Moneymaker’) and the wild currant tomato (*Solanum pimpinellifolium* ‘PI365967’). Through bulked segregant analysis—resequencing, six QTLs were identified. Among them, the major—effect locus qCA6.1 (located on chromosome 6) accounted for 19.28% of the phenotypic variation [42]. Notably, ma1.1, ma3.2, ma3.3, and cca3.3 loci overlapped with those identified by Tieman et al. in heirloom tomatoes. In addition, QTLs controlling MA and CA were co-localized on chromosomes 1, 3, 6, and 7 [43].

For a single major QTL, the heritability of TSS it explains can reach up to approximately 30%, but a more common range is 5% to 15%. For the sum of all known QTLs, they may explain more than 50% of the heritability. There are epistatic interactions among different QTLs controlling the TSS content. This intergenic interaction effect is an important genetic basis for the complex quantitative trait of tomato TSS. A study on processing tomatoes found that there is a positive epistatic effect between the high-TSS loci in the introgression lines IL7-3 and IL9-2-5 derived from *Solanum pennellii*. When these two loci are present simultaneously, they can significantly increase the TSS content of hybrid offspring, showing a synergistic effect [7].

### 2.2. QTL Mapping and Identification Based on GWAS

GWAS combined with high-throughput genotyping technologies have become a key to dissecting the genetic basis of complex traits in plants [8]. The development of next-generation sequencing technologies and the reduction in sequencing costs have jointly transformed GWAS from an expensive technique into a powerful and standardized research tool capable of systematically dissecting the genetic basis of complex traits by promoting the expansion of sample size, the increase in marker density, the extension of research scope, and the enhancement of statistical power [44]. Newly developed mGWAS have further expanded this approach, providing an efficient forward genetics strategy to reveal the genetic and biochemical mechanisms of plant metabolism [45,46,47,48,49,50]. Sauvage et al. conducted an association analysis of metabolites influencing tomato quality using 5995 SNP markers in a natural population of 163 tomato accessions. A total of 8 loci associated with TSS were identified, distributed across chromosomes 2, 3, 6, 7, 8, 9, 11, and 12; 4 loci related to fructose were identified on chromosomes 3, 4, 5, and 6; 3 loci associated with sucrose were mapped on chromosomes 2, 4, and 5; and 2 loci associated with malate were mapped on chromosomes 2 and 6 [51]. Another study precisely identified two key loci controlling glucose and fructose contents: *Lin5* at approximately 31.8 Mb on chromosome 9 (version SL4.0) and *SSC11.1* on chromosome 11, as well as multiple loci controlling organic acids. Notably, a specific Asn366Asp mutation in *Lin5* (which encodes an extracellular invertase) was further highlighted to significantly increase the hexose content in fruits [43]. A GWAS of 92 primary metabolites across 302 tomato accessions identified multiple loci associated with key fruit quality traits, including TSS, fructose, glucose, malate, and citrate. Notably, the major-effect locus TFM6 (tomato fruit malate 6) on chromosome 6, encoding the aluminum-activated malate transporter 9 (*Sl-ALMT9* in tomato), was pinpointed as the primary QTL governing malate variation among genotypes [52]. A GWAS of TSS in red-ripe fruits of 481 tomato germplasm accessions further identified 5 significantly associated QTLs on chromosomes 2, 5, 6, 8, and 9. A sugar transporter, *STP1*, was identified on chromosome 2 as a regulator of TSS [53].

In summary, these GWAS collectively paint a “panoramic view” of the genetic basis of tomato sugar content: it is a complex network controlled by multiple genes, where chromosomes 2, 3, 5 and 9 are the major-effect regions influencing TSS, and several key functional genes have been precisely mapped. Major loci such as *Lin5* and *Sl-ALMT9* have been precisely localized in numerous independent studies. This provides a solid theoretical foundation and valuable target resources for subsequent flavor improvement breeding through molecular marker-assisted selection or gene editing.

## 3. Metabolic and Transport Mechanisms of Main Sugars and Acids in Tomato TSS and Related Genes

### 3.1. Fruit Development and Overview of TSS Composition

The development and ripening of tomato fruits proceeds through three stages: the cell division stage (2–10 d after flowering), during which cells proliferate rapidly and photosynthetic products are mainly allocated to cell division; the cell expansion stage (10–30 d after flowering), during which cell volume increases while soluble sugar content rises slowly and organic acid accumulation peaks; and the ripening stage (from 40 d after flowering onward), during which soluble sugars accumulate rapidly, organic acids are consumed, and TSS content increases significantly [9].

TSS in tomatoes constitutes approximately 60% of their dry weight and is primarily composed of soluble sugars (including glucose, fructose, and sucrose) and organic acids (mainly CA and MA). Among these, fructose and glucose account for 55–65% of TSS [54], while MA and CA together represent approximately 90% of the total acid content [55]. Therefore, the biosynthesis, degradation, and transport of major sugars and acids in tomatoes are of great importance for TSS improvement. Accumulation of TSS involves complex molecular regulatory mechanisms for sugars and organic acids. Studies have indicated that key enzymes in the glycolytic pathway (such as phosphofructokinase) are genetically associated with the levels of CA and MA, indicating that carbon allocation to organic acids directly influences TSS accumulation [56]. Moreover, the *MdNADP-ME* gene has been shown to regulate the coordinated accumulation of both acids and sugars [41]. In modern flavor-oriented breeding, an increase in TSS and sugar content does not necessarily lead to a reduction in acid levels. By precisely selecting and pyramiding specific superior alleles (such as *Lin5*), it is entirely feasible to develop tomato varieties that exhibit both high sugar and high acid content, resulting in a rich and well-balanced flavor profile.

### 3.2. Metabolic and Transport Mechanisms of Sucrose, Glucose, and Fructose and Related Genes

The contents of sucrose, fructose, and glucose in tomato fruits largely determine TSS, which is critical for fruit quality. Wild germplasm provides valuable resources for breeding tomato varieties with high TSS. According to sugar accumulation type, tomatoes can be classified into two categories: hexose-accumulating and sucrose-accumulating. Green-fruited wild tomato species such as *Solanum chmielewskii*, *Solanum hirsutum* and *Solanum hirsutum* f. *glabratum*, *Solanum peruvianum*, and *Solanum neorickii* accumulate sucrose in fruits, whereas cultivated tomato varieties primarily accumulate reducing sugars such as glucose and fructose, along with smaller amounts of sucrose [57].

**Figure 1:**
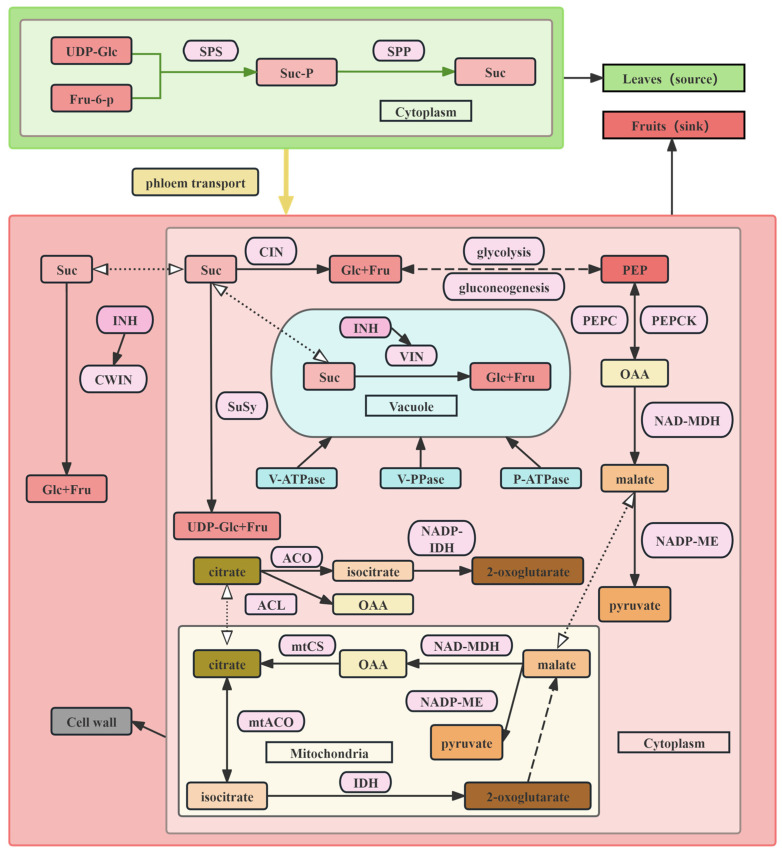
Metabolism and transport of major sugars and acids in tomatoes.

In higher plants, sucrose synthesized in leaves (sources) via photosynthesis is transported to sink organs through the phloem. In sink organs, sucrose is enzymatically degraded into hexoses (Figure 1), supporting organ growth and development [58]. The accumulation of soluble sugars is regulated by a complex network of sugar metabolism, including sugar synthesis, transport, decomposition, and signal transduction. Current research has mainly focused on key enzymes in this network, such as sucrose synthase, sucrose phosphate synthase, and invertase, as well as sugar transporters [59] and some transcriptional regulators, which have significantly advanced the understanding of molecular mechanisms underlying sugar accumulation.

#### 3.2.1. Metabolic and Transport Mechanisms of Sucrose, Glucose, and Fructose

Sucrose metabolism is a crucial component of sugar accumulation, in which SPS, SuSy, and INV play essential roles. SPS plays a crucial role in sucrose accumulation. In many fruits, sucrose accumulation during ripening is closely associated with increased SPS activity. SPS catalyzes the synthesis of sucrose-6-phosphate using uridine diphosphate glucose as the donor and fructose-6-phosphate as the acceptor. Sucrose-6-phosphate is subsequently hydrolyzed into sucrose and phosphate ions by sucrose-6-phosphate phosphohydrolase (SPP). As SPS and SPP exist as a complex in plants, the reaction catalyzed by SPS to produce sucrose is irreversible [11]. SuSy catalyzes the reversible reaction of sucrose metabolism and exerts significant regulatory effects on fruit quality formation and signal transduction [60]. SuSy and INV hydrolyze sucrose into hexoses, and their activities are regulated by invertase inhibitors (INVINH) [61]. INV catalyzes the decomposition of sucrose into glucose and fructose, contributing to processes such as sugar synthesis, degradation, and transport [13]. In cultivated tomato (*Solanum esculentum*), high INV activity results in substantial accumulation of glucose and fructose in vacuoles, while the sucrose content remains low. Conversely, in *Solanum peruvianum* and *Solanum chmielewskii*, vacuolar INV activity is low, leading to higher sucrose accumulation and reduced glucose and fructose contents. Based on pH conditions, INV is divided into AI and neutral invertase (NI). Cell-wall invertase (CWIN) and vacuolar invertase (VIN) belong to AI. The CWIN encoded by the *Lin5* gene on chromosome 9 functions in the intercellular space and cell wall and participates in sucrose decomposition during extracellular unloading from the phloem [36]. VIN, encoded by the *Sucr* gene on chromosome 3, regulates sucrose utilization within vacuoles, affecting sucrose levels, total sugar, and TSS content in fruits [33]. Cytoplasmic invertase (CIN), classified as a neutral invertase [62], functions in cooperation with SuSy to catalyze the hydrolysis of sucrose into glucose and fructose [58].

To gain an in-depth understanding of the regulation of soluble sugar transport in tomato fruits, two homozygous cherry tomato inbred lines, such as “TB0023” (hexose-accumulating) and “TB0278” (sucrose-accumulating), can be analyzed to characterize sugar transport mechanisms. In the phloem unloading pathway, transport may shift from the symplast to the apoplast or proceed through a mixed symplastic–apoplastic route. The high activities of AI, SPS, SuSy, and the sugar transporters LeSUT1, SlSWEET2a, and SlSWEET12c are critical for promoting hexose accumulation, whereas in sucrose-accumulating types, LeSUT2, SPS, SS, and SlSWEET1b/5b/11b/7a/14 play predominant roles [63]. Silencing of acid invertase TIV1 altered the composition of fruit sugars, increasing sucrose content and reducing fructose content [64]. The sucrose transporter (SUT) also regulates phloem unloading capacity. Inhibition of *SUT2* expression can reduce glucose, fructose, and sucrose levels in tomato fruits [65]. Silencing of the plasma membrane-localized proteins *SlSWEET7a* and *SlSWEET14*, which mediate sugar transport, increases fruit sugar content, promotes taller plant growth, and produces larger fruits in *SlSWEET7a*-silenced lines [66]. In contrast, overexpression of the *SlFgr* gene from the *SWEET* family can reduce glucose content and increase fructose content [67]. The heterologous expression of the apple hexose transporter MdHT2.2 in tomato significantly elevates the glucose, fructose, and TSS contents in mature fruits, while decreasing sucrose concentration [68]. Sugar transporters, such as the tonoplast sugar transporter (TST) and the vacuolar glucose transporter (VGT), mediate sugar transfer to vacuoles [69]. The apple cytoplasmic H^+^/glucose symporter MdERDL6-1 promotes glucose efflux and upregulates the H^+^/sugar antiporter genes *TST1* and *TST2*, thereby increasing glucose, fructose, and sucrose accumulation in tomato fruits. In contrast, double mutation of *SlTST1/2* markedly reduces the soluble sugar content [70]. STPs generally function in glucose and fructose transport. CRISPR/Cas9 knockout of *STP1* significantly reduces TSS and soluble sugar content in tomato fruits. Mechanistically, the zinc-finger protein ZAT10-LIKE specifically binds to the insertion-type promoter to enhance expression, whereas this binding capacity is absent in the deletion-type promoter, which explains the genetic basis for decreased TSS in modern cultivated tomatoes [53].

#### 3.2.2. Key Genes and Transcription Factors of Sucrose, Glucose, and Fructose

In recent years, multiple studies have identified key genes involved in sugar metabolism (Table 1). The *AGPase L1* allele of ADP-glucose pyrophosphorylase increases soluble sugar content by enhancing transient starch accumulation [71]. The upregulation of *AGPase* expression in tomatoes enhances the starch and TSS contents, while the reduced expression leads to decreased starch synthesis, delayed flowering, and lower yield [72]. VIFs regulate INV activity by forming inactive complexes. In tomatoes, overexpression of *SlVIF* inhibits sucrose hydrolysis and reduces hexose accumulation, whereas suppression of *SlVIF* promotes sucrose hydrolysis and increases hexose content [73]. As a key gene encoding CWIN, *Lin5* regulates sugar and fructose accumulation in tomato fruits and is controlled by multiple factors. Silencing of *Lin5* markedly reduces glucose and fructose levels [38], whereas INVINH1 can specifically suppress *Lin5* activity, and the vacuolar-processing enzyme *SlVPE5* negatively regulates sugar accumulation [74]. CRISPR/Cas9 editing has provided further insights into gene functions and interactions. Wang et al. suggested that silencing either *SlINVINH1* or *SlVPE5* increased the glucose, fructose, and TSS content, while double knockouts exerted stronger effects, indicating the synergistic regulation [39]. Kawaguchi et al. successfully knocked out *SlINVINH1* using CRISPR/Cas9 and Target-AID technologies, generating high-sugar tomato lines without adverse effects such as reduced fruit size or poor plant growth, thereby confirming the potential of this gene for quality improvement [75]. Studies have shown that knockout of the ripening-specific vacuolar invertase SlVI induces sucrose accumulation, which suppresses the expression of key starch degradation genes *BAM3* and *AMY2* through a negative feedback mechanism, thereby significantly increasing sucrose and TSS contents while enhancing fruit firmness and post-harvest disease resistance [76]. In addition, the calcium-dependent protein kinases SlCDPK27/26 function as the “sugar brake” by phosphorylating SuSy to promote its degradation. The knockout of these kinases can increase glucose and fructose contents by 30%, enhancing sweetness perception without affecting yield [77].

Additionally, several transcription factors have been implicated in the regulation of sugar accumulation (Table 1). The MADS-box transcription factor *RIN* modulates sucrose metabolism and fruit ripening by directly binding to and regulating the expression of *SlVI* and *SlVIF* [73]. The bZIP transcription factors *SlbZIP1* and *SlbZIP2* participate in sucrose-induced translational silencing (SIRT), thereby affecting sugar content in tomato fruits [87]. A dual-gRNA CRISPR/Cas9 system targeting the upstream open reading frame (uORF) of *SlbZIP1* enhances the transcriptional activity of *SlbZIP1* and related genes, leading to significant increases in sugar and TSS contents, with edited lines exhibiting improved fruit traits and normal growth [80]. The NAC transcription factor *SlNAP2* responds to ABA signaling and regulates ABA homeostasis through feedback, delaying leaf senescence and enhancing soluble sugar and TSS accumulation [78]. The auxin response factor *SlARF10* positively regulates starch and sucrose accumulation by modulating the expression of *AGPase*, a key gene in starch biosynthesis [79]. A recent study has reported that heterologous overexpression in tomato reveals that the nuclear-localized transcription factor *MdWRKY126* can coordinately upregulate sucrose synthesis and transport genes (*SPS* and *SUT*) and suppress sucrose decomposition genes (*CWINV* and *SuSy*) as well as hexose transport genes (*HTs* and *TSTs*) [84]. The tomato C2H2-type zinc finger transcription factor SlC2H2-71 is highly expressed in low-TSS fruits. The knockout of *SlC2H2-71* resulted in increased TSS, fructose, glucose, MA, and CA contents in red-ripe tomato fruits, accompanied by a reduction in sucrose content. Further analysis demonstrates that *SlC2H2-71* can negatively regulate soluble solid accumulation in red ripe fruits by directly binding to the *SlLIN5* promoter and repressing its expression [82].

### 3.3. Metabolic and Transport Mechanisms of Malic Acid and Citric Acid and Related Genes

#### 3.3.1. Metabolic and Transport Mechanisms of Malic Acid and Citric Acid

The biosynthesis and degradation of MA are associated with multiple metabolic pathways, including the respiratory process, TCA cycle, and glyoxylate cycle. During anaerobic glycolysis, oxaloacetate (OAA), generated by the carboxylation of phosphoenolpyruvate (PEP) in the cytoplasm via phosphoenolpyruvate carboxylase (PEPC), is reduced to MA [88]. Conversely, NAD-dependent malate dehydrogenase (NAD-MDH) oxidizes MA to OAA, which is subsequently decarboxylated by phosphoenolpyruvate carboxykinase (PEPCK) to regenerate PEP. This process is associated with gluconeogenesis, that is, the conversion of organic acids to sugars. In addition, MA can be decarboxylated to pyruvate by NADP-dependent malic enzyme (NADP-ME) (Figure 1) [89,90,91,92]. Malate produced in the cytosol can be transported into mitochondria to participate in the TCA cycle, where malate and citrate interconversion occurs. Mitochondrial citrate synthase (mtCS) directly regulates citrate synthesis in OAA. Citrate produced in mitochondria can be reversibly converted to isocitrate by mitochondrial aconitase (mtACO) and then degraded to α-ketoglutarate by isocitrate dehydrogenase (IDH) [14,15]. In the cytoplasm, ATP citrate lyase (ACL) cleaves citrate into OAA and acetyl-CoA, whereas cytosolic aconitase (cytACO) and NADP-dependent isocitrate dehydrogenase (NADP-cytIDH) catalyze α-ketoglutarate formation, which feeds into the GABA pathway (Figure 1) [14,93].

In addition to metabolism, the transport of organic acids into vacuoles is essential for the regulation of fruit acidity. Once malate and citrate are transported into vacuoles, protonation maintains electrochemical gradients of MA and CA across the tonoplast, driving their continuous accumulation [14]. This process relies on vacuolar pH and membrane potential, which can be generated by proton pumps that transport protons into vacuoles. Three types of proton pumps, including V-ATPase, V-PPase, and P-type ATPase, participate in organic acid accumulation [41]. The aluminum-activated transporter *SlALMT9* is a key protein controlling MA accumulation in tomato fruits, and variation in its promoter region has been linked to disruption of *WRKY* transcriptional repressor binding, leading to elevated MA levels [85]. Polymorphisms near the *SlALMT9* locus have also been associated with variations in the MA content [94]. In addition, an InDel located upstream of *Solyc06g072840*, which encodes hydrogen peroxide-induced protein 1, has been linked to the MA content [95].

#### 3.3.2. Key Genes and Transcription Factors of Malic Acid and Citric Acid

Organic acid metabolism as a key determinant of tomato flavor quality is regulated by multiple genetic factors (Table 1). Association mapping in tomato has identified loci for MA, CA, total organic acids, and pH on nearly every chromosome [96,97,98]. GWAS on MA has identified numerous SNPs, including 15 high-confidence annotated genes based on linkage disequilibrium and lead SNPs [96]. In the GWAS based on SNPs, InDels, and structural variations (SVs), 11 candidate genes associated with citrate content were identified [99]. Furthermore, silencing of *PEPCK* decreased gene expression and enzyme activity, reduced starch synthesis, and altered metabolite levels, including decreased fructose and CA, along with increased MA [86]. Overexpression of the ABA response factor *SlAREB1* can significantly enhance organic acid accumulation, with elevated CA and MA contents through the upregulation of genes such as citrate synthase, confirming the role of ABA-mediated regulation in organic acid metabolism [51].

## 4. Effects of Environmental Factors on the TSS Content of Tomato Fruits

The cultivation environment exerts a significant influence on the TSS content of tomatoes. Therefore, regulating growth conditions through various approaches is critical for enhancing TSS in tomato fruits.

### 4.1. Temperature and Light

Temperature and light in the cultivation environment have a significant influence on tomato quality. Reductions in temperature and light lead to decreases in vitamin C (VC), TSS, soluble protein, and lycopene, while organic acid content increases [100]. Light intensity directly affects TSS levels in tomato fruits, where daytime lighting increases TSS by 8.2% in summer and 24% in winter, and LED cross-illumination at night during winter enhances TSS by 20%, while no significant effect is observed in summer [101]. Appropriate combinations of light qualities promote tomato growth and development. Combined red, blue, and white light (R1W1B0.5) significantly enhances photosynthesis, biomass, and fruit quality by activating the photoreceptor module (*SIPHBYB1*, *SlCRY1*, *SlHY5*, *SlLHCA/B* and *SlCYCB*) [102]. Under 16 h photoperiod conditions, treatment with combined red-blue light (R1B0.8) compared with white light can markedly increase chlorophyll content and biomass. This light spectrum enhances light capture and electron transfer efficiency by upregulating genes encoding photosystem subunits (*SlPsaA/B/C*) and the light-harvesting complex (*SlLHCB/A*), thereby improving the photosynthetic rate and elevating fructose and glucose levels [103]. The light systemic signal ELONGATED HYPOCOTYL 5 (HY5) directly activates sucrose metabolism genes, including *LIN5*, *LIN6*, *VI*, *SS1* and *SS7*, by binding to cis-acting promoter elements. Fructose, glucose, and TSS contents were significantly increased in fruits of the *HY5* overexpression line OE-HY5 [83].

### 4.2. Water and Nutrients

Agronomic measures can effectively and coordinately improve tomato fruit flavor by regulating key physiological processes and expression of sugar- and acid-related genes. Water deficit (WD) irrigation has been reported to promote the accumulation of reducing sugars, total acids, vitamin C, and TSS in fruits, thereby enhancing sweetness and enriching flavor [104]. A total of 5% biochar combined with 60% crop evapotranspiration deficit irrigation was an effective strategy to optimize tomato planting management under saline irrigation (2.3 dS m^−1^). This strategy can not only save 20–40% irrigation water, but also significantly improve fruit quality (soluble solids and total sugar content up to 99.75%), and reduce fruit sodium content, thus achieving the dual goal of “water saving and quality improvement” [105]. Potassium application has also been shown to improve fruit quality by increasing concentrations of TSS, sucrose, fructose, glucose, CA and MA. This improvement was achieved by upregulating the expression of *SlSWEETs* and *SlSUTs*, thereby promoting sucrose metabolism through the activation of *SlSPS1*, *SlSUSs*, and *SlTINVI*, which ultimately increased soluble sugar accumulation [106]. As a macronutrient, Cl^−^ plays an important role in plant growth. Treatment with 3–5 mM Cl^−^ at the red-ripe stage increased glucose by 18.6%, fructose by 10.7%, and TSS by 9.4% in tomato fruits without affecting yield. Additionally, Cl^−^ enhances the activities of α-amylase, sucrose phosphate synthase, and sucrose synthase, thereby strengthening photosynthesis [107].

### 4.3. CO_2_ and Exogenous Substances

Compared with 400 ppm ambient CO_2_, Micro-Tom tomato fruits exposed to 900 ppm CO_2_ significantly decreased sucrose content and significantly increased fructose and glucose content, with the most statistically significant increase in glucose. This suggests that high concentrations of CO_2_ alter the sugar composition of tomato fruit [108]. The study confirmed that composting by using crop residues and animal manure in greenhouses can be used as an effective and inexpensive method of CO_2_ enrichment, doubling the CO_2_ concentration in the greenhouse during the day, increasing the yield of cherry tomatoes by up to 38%, and significantly increasing the concentration of TSS and soluble sugar [109]. Furthermore, pre-harvest spraying with GABA markedly enhances tomato fruit quality through metabolic regulation. The effect was most pronounced 3 d after application. Exogenous GABA enters cells via the GABA transporter, induces endogenous GABA metabolism, and promotes the accumulation of organic acids while upregulating related genes. In addition, GABA treatment regulates sugar metabolism by inhibiting part of glycolysis, increasing trehalose 6-phosphate (Tre6P) content, and elevating glucose, fructose, and sucrose levels [81].

## 5. Approaches to Increase TSS in Tomato Fruits

### 5.1. Cross Breeding

Improving TSS is an important breeding objective for tomatoes. Cross breeding remains a key approach for enhancing TSS by crossing cultivated tomatoes with high-TSS germplasms. This method is simple, easy to apply, and widely accepted because it does not involve exogenous gene introduction. However, this approach has significant limitations for improving the complex quantitative traits of TSS. The selection of high-TSS phenotypes relies primarily on phenotypic evaluations, such as refractometer measurements, and can only be performed after fruit maturity. This results in prolonged breeding cycles, low efficiency [16], and limited accuracy, as outcomes are strongly influenced by environmental factors, thereby obscuring true genetic potential. Moreover, the introduction of favorable alleles for high TSS is often accompanied by the incorporation of unfavorable alleles linked to them, such as those associated with reduced fruit size.

### 5.2. MAS and GS

Forward genetics has advanced our understanding of the genetic basis of phenotypic traits and revealed natural genetic variation. MAS utilizes relevant DNA markers to track traits of interest, providing an effective strategy for early selection at the juvenile stage and accelerating breeding [110]. MAS requires the identification of loci associated with traits, which is typically achieved through QTL mapping or linkage analysis [18]. In tomato TSS research, representative markers have been developed, including the *STP1* InDel_21 marker related to sugar transport [53] and the InDel-based cleaved amplified polymorphic sequence (CAPS) marker of *SlALMT9* related to malate transport [111]. However, considerable work remains to be conducted from QTL discovery to the development of reliable markers and their application in MAS, including the validation of marker predictive power for traits. GS, which can be used to evaluate complex traits using whole-genome DNA markers, has proven to be more effective than MAS. Substantial reduction in genotyping costs has made GS an attractive breeding strategy. However, its low prediction accuracy remains a critical limitation. GS prediction accuracy in tomato breeding varies with trait types, population structures, and marker densities. For simple traits with high heritability (such as disease resistance), the GS prediction accuracy can usually reach 0.7–0.8; for complex quantitative traits (such as yield and soluble solid content), the accuracy is generally between 0.5 and 0.7. Improving accuracy through the development of advanced statistical machine learning models and optimization of training populations represents a promising yet challenging direction for enhancing genetic prediction and facilitating the broader application of GS in breeding programs [19]. The successful application of GS in the research on resistance to bacterial wilt in tomato [112], highlights its potential to facilitate early selection of tomato TSS traits and promote more efficient breeding.

### 5.3. Genome Editing

With the development of genome editing technologies, tools such as CRISPR/Cas9 can be employed to efficiently knock out, knock in, or perform base editing of specific genes in the tomato genome, thereby creating new germplasms, accelerating the breeding process, and overcoming interspecific hybridization barriers [113]. To address the yield reduction under the global warming and the shortage of heat-tolerant varieties (expected to intensify by 3–13%), researchers have proposed the climate-responsive carbon allocation sink optimization (CROCS) strategy. Using prime editing technology, a 10 bp heat shock element (HSE) was precisely inserted into the promoter of CWIN gene *Lin5*. Following this modification, CWINs exhibited heat-responsive upregulation under both experimental and field conditions, significantly enhancing carbon allocation to fruits. Tests have demonstrated that the yield per mu of tomato AC increases by 33%, and the fruit sugar content increases [114]. The first genome-edited crop to reach the market was a tomato enriched in GABA, named Sicilian Rouge High GABA [20]. In this variety, the calmodulin-binding domains of the glutamate decarboxylase (GAD) genes *SlGAD2* and *SlGAD3* can be knocked out to increase GAD activity, thereby enhancing the conversion of glutamate to GABA and improving the nutritional quality of tomato fruits [115]. On the basis of gene editing, design-based breeding integrates genomics, bioinformatics, and genetic design strategies to achieve systematic analysis and precise assembly of breeding targets. This approach promotes transformation of the breeding process from “empirical screening” to “targeted design”, thereby improving both the predictability and efficiency of breeding.

### 5.4. Model-Based Precision Cultivation

By establishing crop models, the interactions between environmental factors and the physiological and biochemical processes of tomato growth and development can be better investigated. Zhou et al. [116] developed an integrated model of tomato plant and fruit growth combined with tomato growth and fruit sugar metabolism (TGFS). Further scenario-based simulations indicated that reducing nitrogen fertilizer by 15–25% and irrigation by 10–20% relative to the current level would increase tomato fresh weight by 27.8–36.4% and soluble sugar concentration by 10% [117]. A process-based biophysical model of fleshy fruit development [116] combined with an enzyme-based kinetic model of sugar metabolism [118] predicted that tomatoes could be larger and sweeter if biophysical factors and transmembrane transport were optimized during fruit development [119].

In brief, hybrid breeding is widely applied but relies on phenotypic selection, with limitations such as long cycles, low efficiency, and linkage drag (e.g., smaller fruit size). MAS enables early selection using DNA markers, yet it depends on accurate prior QTL mapping. GS improves complex traits through whole-genome prediction, and its prediction accuracy is the current key bottleneck for application. Gene editing can precisely design genes and break linkage drag, and has successfully bred tomatoes with heat tolerance, increased yield, and high GABA content. Precision cultivation optimizes environmental management through growth models and can effectively improve TSS without changing the variety. Gene-edited crops are generally regarded as a new type of breeding technology, and their regulatory policies are still being continuously improved and clarified. It is necessary to closely monitor the latest relevant regulations issued by the Ministry of Agriculture and Rural Affairs to ensure the compliance of breeding methods. In summary, improving tomato TSS no longer relies on a single technology. The future direction lies in technology integration: combining the macroscopic prediction ability of GS with the precise design ability of gene editing, and using MAS to rapidly introgress known major genes. By integrating model simulation and optimized management, the genetic potential of varieties can be maximized.

## 6. Conclusions and Future Prospects

As a core quantitative trait for evaluating fruit flavor and quality, TSS in tomatoes can be regulated by multiple genes and is strongly influenced by environmental conditions. The extremely high TSS levels in wild tomato resources (approximately two to three times those of cultivated varieties) provide valuable genetic material for identifying key loci. Multiple major QTLs and key genes regulating TSS have been identified through linkage analysis using interspecific hybrid populations or GWAS with natural populations. A single major QTLs (*Lin5*, etc.) can explain up to ~30% of TSS heritability, though 5–15% is more typical. Collectively, known QTLs account for over 50% of heritability. With the widespread application of next-generation sequencing and genotyping technologies, additional regulatory genes and molecular markers associated with sugar and acid metabolism have been gradually revealed, laying the foundation for MAS and GS.

The pathways to enhance tomato TSS through environmental regulation are multiple and feasible. Modern cultivation management has changed from single factor control to multi-factor coordinated regulation, the core of which is to influence plant physiological process and gene expression network by regulating environmental signals, and finally realize coordinated and optimized accumulation of flavor substances such as sugar and acid.

In modern agriculture, alternative crop improvement strategies beyond hybridization have been proposed, including mutation breeding, genomic engineering, and genome editing [120]. Chemical mutagens and irradiation can induce random mutations [121], but the low frequency of beneficial variants and difficulty in identifying desirable individuals remain challenges [122]. Continuous advances in plant biotechnology aim to address these issues [123]. Currently, tomato breeding has shifted from cross breeding to precision design breeding, integrating genome editing and multi-omics approaches. The CRISPR/Cas system enables precise knockout, knock-in, or base editing of target genes, mimics natural variations without introducing exogenous DNA, and significantly improves breeding efficiency and safety. Studies have identified major genes (*Lin5*, *Sl-ALMT9*, etc.) that regulate the sugar and acid contents in tomatoes. Furthermore, by knocking out SlINVINH1 through gene editing, a successful approximately 30% increase in sugar content has been achieved while maintaining normal fruit size. The application of genome editing technology in the “de novo domestication” of wild tomatoes has been particularly notable [124,125,126]. To increase the TSS in tomato, the era of “combination breeding” has arrived. Future winners will not be determined by the mastery of a single technique but by the ability to seamlessly integrate technologies such as gene editing, marker-assisted selection, and genomic selection to establish a rapid breeding system spanning from the laboratory to the dinner table.

With the advancement of sequencing technologies and the construction of pangenome maps for more tomato germplasm resources, by integrating multi—dimensional datasets such as transcriptomics, metabolomics, and proteomics, and leveraging artificial intelligence analysis, pan-genomics can systematically decipher the complex regulatory networks of tomato TSS by uncovering the entire genetic diversity of the tomato species. This approach enables researchers to discover novel genes and pathways that were previously hidden and to identify key genetic nodes for precise breeding. High-throughput TSS measurement under phenomics automation serves as a bridge connecting genotypes and trait manifestations. It has revolutionized the way we perceive and screen fruit quality through the integration of spectroscopy, automated platforms, and data analysis. In future breeding or cultivation practices, the “beneficial microbial community conducive to the formation of high quality” can be considered as a potential screening indicator to select tomato varieties with higher symbiotic efficiency with beneficial microorganisms [127].

Future research should focus on four aspects: the application of genome editing and related technologies to break the negative genetic correlation between TSS and yield; an in-depth analysis of the sugar-acid metabolic network and its responses to environmental factors, thereby balancing TSS, stress resistance, and adaptability through multi-trait collaborative selection; the optimization of the sugar-acid ratio in line with consumer preferences, while enhancing volatile flavor substances to achieve improved tomato flavor; and the continuous integration of multiple technologies with mechanistic analysis to promote directional breeding and industrial application of tomato varieties with high TSS.

## Figures and Tables

**Table 1:** Functions of genes and transcription factors regulating the main sugar and acid components in the formation mechanism of tomato TSS.

Target Traits	Target Genes	Effect	References
Sugar	*TIV1*	Inhibition of gene expression increases sucrose content and reduces fructose content in fruits.	[64]
Sugar	*SUT2*	Inhibition of gene expression decreases contents of glucose, fructose, and sucrose in tomato fruits.	[65]
Sugar	*Lin5*	Gene silencing significantly reduces glucose and fructose contents in tomato fruit.	[38]
Sugar	*AGPase*	Upregulation of gene expression increases soluble sugar content.	[72]
Sugar	*SlVIF*	Overexpression reduces sucrose accumulation and hexose content, whereas suppression increases hexose accumulation.	[73]
Sugar	*RIN*	Regulation of the vacuolar invertase gene *SlVI* and its repressor *SlVIF* controls sucrose metabolism and fruit ripening.	[73]
Sugar	*SlNAP2*	Suppression of gene expression increases soluble sugar and TSS levels.	[78]
Sugar	*SlFgr*	Overexpression decreases glucose content but increases fructose content.	[67]
Sugar	*SlARF10*	Overexpression significantly increases starch, fructose, and sucrose accumulation in fruits.	[79]
Sugar	*MdHT2.2/SlLIN5*	Allogeneic expression of *MdHT2.2* reduces sucrose concentration but increases glucose and fructose. Deletion of *SlLIN5* reduces glucose, fructose, and TSS contents while increasing sucrose concentration.	[68]
Sugar	*SlINVINH1/SlVPE5*	Deletion of *SlINVINH1* or *SlVPE5* increases glucose, fructose, and TSS contents. Double deletion further enhances these contents.	[39]
Sugar	*SlSWEET7a/SlSWEET14*	Gene silencing increases sugar content in ripe fruits.	[66]
Sugar	*MdERDL6-1*	Overexpression increases glucose, fructose, and sucrose contents.	[70]
Sugar	*SlTST1/SlTST2*	The double mutant lines of *SlTST1* and *SlTST2* decrease glucose by 40%, fructose by 50%, sucrose by 50%, and TSS by 40%.	[70]
Sugar	*SlbZIP1*	Deletion of inhibitory region enhances hexose and TSS contents.	[80]
Sugar	*STP1*	Gene knockout reduces TSS, glucose, fructose, and sucrose contents.	[53]
Sugar	*SlVI*	Gene knockout increases sucrose and TSS contents in fruits.	[81]
Sugar	*SlCDPK27/26*	Gene knockout increases glucose and fructose levels by 30%.	[77]
Sugar; Organic acid	*SlC2H2-71*	Gene knockout increases TSS, fructose, glucose, MA, and CA contents while decreasing sucrose content.	[82]
Sugar	*HY5*	Overexpression increases fructose, glucose, and TSS contents.	[83]
Sugar	*MdWRKY126*	Overexpression promotes sucrose accumulation and reduces hexose accumulation.	[84]
Organic acid	*SlAREB1*	Overexpression increases organic acid accumulation.	[55]
Malic acid	*SlALMT9*	Release of transcriptional repression promotes high accumulation of MA in fruits.	[85]
Organic acid	*PEPCK*	Gene silencing increases the malic acid content while reducing the glucose and fructose contents in fruits.	[86]

## Data Availability

No new data were created or analyzed in this study. Data sharing is not applicable to this article.

## Abbreviations

The following abbreviations are used in this manuscript:

ACL	ATP citrate lyase
AI	acid invertase
CA	citric acid
CAPS	cleaved amplified polymorphic sequence
CIN	cytoplasmic invertase
CROCS	Climate-responsive optimization of carbon partitioning to sinks
CWIN	cell-wall invertase
cytACO	cytosolic aconitase
GABA	γ-aminobutyric acid
GAD	glutamate decarboxylase
GS	Genomic selection
GWAS	Genome-wide association studies
HSE	heat shock element
HY5	ELONGATED HYPOCOTYL 5
IDH	isocitrate dehydrogenase
INV	invertase
INVINH	invertase inhibitors
MA	malic acid
MAS	marker-assisted selection
mGWAS	metabolome-wide association studies
mtACO	mitochondrial aconitase
mtCS	mitochondrial citrate synthase
NAD-MDH	NAD-dependent malate dehydrogenase
NADP-ME	NADP-dependent malic enzyme
NI	neutral invertase
OAA	oxaloacetate
PEP	Phosphoenolpyruvate
PEPC	Phosphoenolpyruvate carboxylase
PEPCK	phosphoenolpyruvate carboxykinase
QTL	quantitative trait locus
SIRT	sucrose-induced translational silencing
SNP	single nucleotide polymorphisms
SPP	sucrose-6-phosphate phosphohydrolase
SPS	sucrose phosphate synthase
SSR	simple sequence repeats
SuSy	synthase
SVs	structural variations
TCA cycle	tricarboxylic acid cycle
Tre6P	trehalose 6-phosphate
TSS	Total soluble solids
TST	Tonoplast sugar transporter
uORF	upstream open reading frame
VC	vitamin C
VGT	Vacuolar glucose transporter
VIN	vacuolar invertase
